# COVID-19 and APOL-1 High-Risk Genotype-Associated Collapsing Glomerulonephritis

**DOI:** 10.1155/2021/3737751

**Published:** 2021-08-05

**Authors:** Sasmit Roy, Srikanth Kunaparaju, Narayana Murty Koduri, Vikram Sangani, Mytri Pokal, Venu Madhav Konala, Mamtha Balla, Sreedhar Adapa

**Affiliations:** ^1^Lynchburg Nephrology Physicians, 2091 Langhorne Road, Lynchburg, VA 24501, USA; ^2^Richmond Nephrology Associates, 671 Hioaks Road, Suite B, Richmond, VA 23225, USA; ^3^Great Plains Health, 601 W Leota St, North Platte, NE 69101, USA; ^4^Quantum HC, Department of Internal Medicine, Navicent Health, 777 Hemlock Street, Macon, GA 31201, USA; ^5^Precision Cancer Center, 122 St Christopher Dr, Ashland, KY 41101, USA; ^6^Promedica Toledo Hospital, 2142 N Cove Blvd, Toledo, OH 43606, USA; ^7^Adventist Medical Center, 115 Mall Drive, Hanford, CA 93230, USA

## Abstract

Coronavirus Disease 2019 (COVID-19) caused by severe acute respiratory syndrome coronavirus 2 (SARS-CoV-2) primarily affects the lungs and can lead to acute respiratory distress syndrome (ARDS). The ongoing global pandemic has created healthcare and economic crisis for almost every nation of the world. Though primarily affecting the lungs, it has also affected the kidney in various ways including acute kidney injury (AKI), proteinuria, and hematuria. It has been increasingly shown that African American (AA) individuals affected with COVID-19 and presenting with AKI and nephrotic-range proteinuria are very susceptible to focal segmental glomerulosclerosis (FSGS). The APOL-1 gene, associated with the African American population, has been increasingly recognized as a risk factor for FSGS affected with COVID-19. Our case highlights a similar case of COVID-19 in a 65-year-old AA descendant with biopsy-proven FSGS and genetically confirmed APOL-1 alleles.

## 1. Introduction

Coronavirus Disease 2019 (COVID-19) caused by severe acute respiratory syndrome coronavirus 2 (SARS-CoV-2) primarily affects the lungs and can lead to acute respiratory distress syndrome (ARDS). The kidney is another major organ affected by the virus, which can cause acute kidney injury (AKI), proteinuria, and hematuria which are the independent predictive factors for mortality [1, 2]. COVID-19 infects disproportionately minorities and people with low socioeconomic status resulting in an increased risk of hospitalization and higher mortality [3].

We describe a case report of an African American male admitted with fever and shortness of breath and was diagnosed with COVID-19, who developed worsening renal function and proteinuria. The patient was subsequently started on hemodialysis and underwent kidney biopsy.

The kidney biopsy revealed collapsing glomerulonephritis, a rare entity that has reemerged in African American patients (who have a high-risk APOL1 gene), who were infected with COVID-19.

## 2. Case Presentation

A 65-year-old African American male was admitted to the hospital with chief complaints of fever and shortness of breath. The patient was exposed to a family member who has tested positive for COVID-19 and was subsequently confirmed with SARS-CoV-2 infection by reverse transcriptase polymerase chain reaction (RT-PCR) analysis. The patient's past medical history was significant for hypertension, diabetes mellitus type 2, hyperlipidemia, and chronic kidney disease stage 3A with the baseline glomerular filtration rate (GFR) of 50 mL/min. Home medications included amlodipine 10 milligrams (mg) daily, furosemide 40 mg daily, metformin 500 mg daily, and atorvastatin 40 mg daily. Physical examination was remarkable for a temperature of 101 degrees Fahrenheit, pulse rate of 89 beats/min, blood pressure of 108/75 mm Hg, and a respiratory rate of 22 breaths/min. The patient was in moderate respiratory distress using accessory muscles, and the auscultation of the lungs was significant for bilateral (b/l) wheezing and diffuse crackles. The rest of the physical examination was insignificant. The laboratory data are detailed in Table 1 (all admission) and Table 2 (progress in hospital up to 6 weeks after discharge, renal parameters).

The renal function was worse from baseline with the serum creatinine of 6.5 mg/dl, blood urea nitrogen (BUN) of 86 mg/dl, and spot urine protein-creatinine (UPC) ratio of 15.8 gm/gm (baseline values: creatinine 1.2 mg/dl, BUN 32 mg/dl, and UPC 2gm/gm 6 months ago). Renal ultrasound revealed b/l enlarged kidneys, with no hydronephrosis. Although he had nephrotic-range proteinuria, he did not present with other classic features of nephrotic syndrome such as severe albuminemia (i.e., serum albumin <2.5 mg/dl) or peripheral edema. Ocular examination for common diabetic retinopathy features such as glaucoma, macular edema, or microaneurysm were absent. The chest X-ray revealed diffuse infiltrates in b/l lung fields consistent with COVID-19. Other serologies including antinuclear antibody (ANA), complements (C3 and C4), antineutrophil cytoplasmic antibodies (ANCAs), antiglomerular basement membrane antibody, and antiphospholipase A2 receptor (anti-PLA2R) antibodies were normal. Serologies for hepatitis B and C virus, Human Immunodeficiency Virus (HIV), Epstein–Barr Virus (EBV), Cytomegalovirus (CMV), and Parvovirus B19 were all negative.

Serum immunofixation did not reveal any monoclonal protein. The patient was initially started on high-flow oxygen through a nasal cannula and was subsequently started on hemodialysis for worsening renal functions with oliguria on day five of admission.

**Figure 1:**
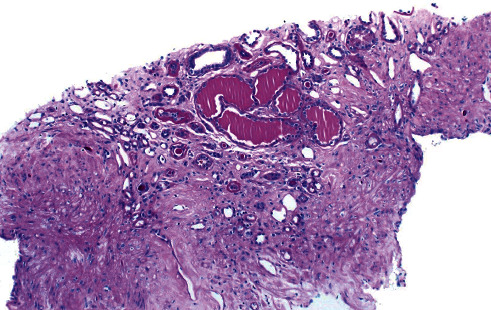
Focal microcystic tubular dilatation.

**Figure 2:**
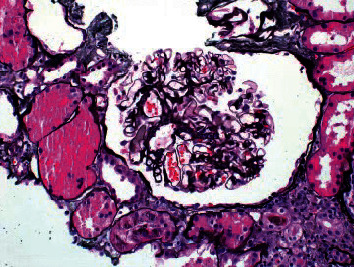
No proliferative glomerular changes.

**Figure 3:**
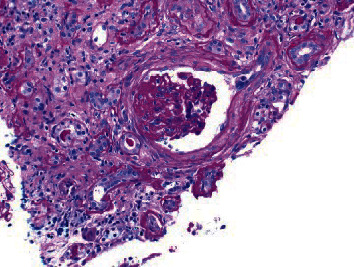
Segmental sclerosis and periglomerular fibrosis with tuft collapse.

**Figure 4:**
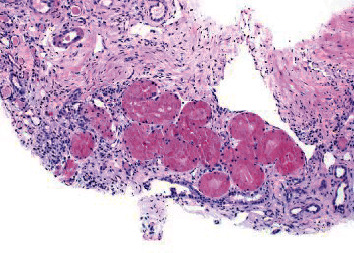
Tubules with protein resorption droplets.

**Figure 5:**
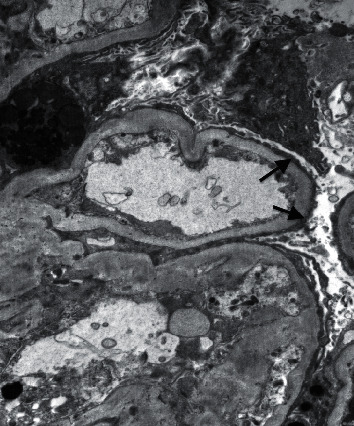
Electron microscopy showing diffuse foot process effacement (arrow).

The patient underwent renal biopsy for proteinuria and AKI to determine the etiology. Kidney biopsy revealed changes consistent with collapsing glomerulopathy, and light microscopy revealed intact glomeruli showing focal segmental sclerosis, associated with loop wrinkling, periglomerular fibrosis, and focal segmental podocyte hyperplasia with tuft collapse. No foci of endocapillary hypercellularity, crescent formation, or necrosis were identified. Out of the five glomeruli in light microscopy, one had global glomerulosclerosis, thereby suggesting roughly 20% renal tissue had global sclerosis. Around 50% globally showed collapsing lesions. The tubular epithelial cytoplasm demonstrated extensive protein resorption droplets, with the presence of focal microcystic dilatation. Interstitium revealed mild mixed inflammatory infiltrates with severe interstitial fibrosis, along with severe arterial intimal fibrosis and mild significant hyaline arteriosclerosis. Immunofluorescence did not show any significant immune deposits, while electron microscopy showed severe effacement of the podocyte foot process. No immune-type electron-dense deposits were identified on electron microscopy. Light and electron microscopy images are provided in Figures 1–5. In situ hybridization for SARS-CoV-2 was negative. Features of diabetic nephropathy such as increase in the mesangial matrix and cell content or decrease in the capillary lumen space were lacking though mild thickening of the glomerular capillary basement membrane was present.

The patient had two APOL1 risk variants detected through genetic testing which revealed compound heterozygosity for APOL1 G1 and G2 risk alleles.

The patient was subsequently treated with hydroxychloroquine, remdesivir, corticosteroids, and intravenous antibiotics during the hospital stay. The patient never needed invasive ventilation support nor intensive care unit (ICU) stay. He was discharged to the outpatient dialysis clinic on day 17 as he continued to require dialysis support. The renal function improved after six weeks, and he was taken off dialysis as his serum creatinine improved to 2.4 mg/dl with no further oliguria.

The patient continues to remain dialysis free till date, and his GFR has improved back to his baseline (pre-COVID-19 state), with the resolution of hematuria and proteinuria (Table 2). For immunosuppression, he completed a three-month course of oral prednisone with gradual tapering.

## 3. Discussion

The involvement of the kidney by COVID-19 is frequent and manifests as acute kidney injury, proteinuria, and hematuria often progressing to End-Stage Renal Disease (ESRD) requiring dialysis. Incidents of AKI, proteinuria, and hematuria in COVID-19 patients are 10%, 40%, and 26%, respectively [1, 2].

Patients with glomerulonephritis usually have hematuria, sustained proteinuria composed largely of albumin. There has been emerging evidence of collapsing glomerulopathy on kidney biopsy in genetically susceptible individuals with African ancestry, infected with COVID-19 [4, 5].

Collapsing glomerulopathy presents as segmental or global glomerular collapse with a rapid deterioration in renal function. Focal segmental glomerulosclerosis (FSGS) refers to a pattern of renal injury characterized by segmental glomerular scars that involves some, but not all, glomeruli, clinically largely manifested as proteinuria. FSGS is classified based on a variety of morphologies on the biopsy finding. Among them, collapsing glomerulopathy (CG) represents an aggressive variant that is characterized by a collapse of the glomerular tuft along with hyperplasia and hypertrophy of the podocytes [6]. Acute tubular injury, inflammation of the interstitium, and tubular dilatation with accompanying microcysts are the commonly associated findings of CG [6].

FSGS is more commonly seen in African Americans. Infections such as HIV, CMV, EBV, and Parvovirus B19 are commonly associated with CG. Malignancies, thrombotic microangiopathy, sickle cell disease, cholesterol embolization, genetic mutations, and certain drugs such as heroin, calcineurin inhibitors, pamidronate, and interferon can also cause CG. It is also found in inflammatory and autoimmune conditions such as hemophagocytic syndrome and systemic lupus erythematous [6].

Polymorphism in the gene-encoding Apolipoprotein L1 (APOL1) is a major risk factor for nearly 10% of African Americans with nondiabetic ESRD, particularly FSGS. APOL1 alleles G1 and G2 inherited from each parent are required to increase the risk of kidney disease when compared to G0 which is considered as a low-risk allele [7]. Although, the exact mechanism on how these high-risk alleles transform the podocytes remains elusive [6]: mitochondrial dysfunction, endosome alteration, activation of protein kinases, cell membrane cation channel dysregulation, and interference with actinomycin in podocytes are the multitude of cellular mechanisms put forth based on the cell culture and transgenic mice studies [6].

COVID-19 has been found to infect a disproportionate ratio of minorities and people of low socioeconomic background leading to increased hospitalization burden and higher mortality rate in these individuals [3]. Based on their study that included combined statistical areas (poverty prone/low − income areas as per the US office of Management and Budget) in 10 major US cities such as New York City, New Orleans, Atlanta, and Chicago, they found 63.5% of the confirmed COVID-19 cases (834126 of 1312679) in the United States as of May 10, 2020, were from those areas itself. In counties with high poverty (median income range, $36,850–$88,960), those with substantially nonwhite populations had an infection rate nearly eight times that of counties with substantially white populations (RR, 7.8; 95% CI, 5.1 to 12) and a death rate more than nine times greater (RR, 9.3; 95% CI, 4.7–18.4) [3].

Surprisingly, it has been found that, patients who had moderate pulmonary symptoms or who recovered from COVID-19, developed AKI and proteinuria and the AKI did not recover despite improvement in respiratory symptoms. This suggests that the effect on the lungs and kidneys are discrete [6]. Despite the initially proposed mechanism of direct renal cell infection [8–10], viral cytotoxic effect on podocyte contributing to AKI based on postmortem studies [6], and electron microscopy demonstrating possible viral particles in patients with CG [11], the follow-up case reports failed to demonstrate virus in a patient with CG by in situ hybridization studies or electron microscopy [6] or RT-PCR essay on renal tissue [12], suggesting an indirect effect of the virus on the kidneys.

Kudose et al. studied 17 patients with COVID-19-related AKI and their kidney biopsies and concluded that AKI in COVID-19 patients could be secondary to cytokine-mediated effects and heightened adaptive immune response but not related to direct viral injury [13]. Another multicentric study showed Acute Tubular Necrosis (ATN) and endothelial injury/thrombotic microangiopathy as a pathological process apart from collapsing GN [14].

SARS-CoV-2 enters ACE2 receptors located in proximal tubules and podocytes in kidneys per postmortem histology [7, 15]. In addition to the above mentioned mechanisms, imbalanced RAS activation, hypovolemia, high PEEP, and hemodynamic changes are suggested by Ahmadian et al. [16] Also, IgA nephropathy was seen as a causative agent for AKI in COVID-19 in one case [17].

### 3.1. Treatment

Treatment of COVID-19 is still an evolving process with various guidelines and studies conducted worldwide [18]. Treatment of secondary FSGS involves management of the etiology and reduction of proteinuria. The benefit of remdesivir is not observed in patients with eGFR <30, though our patient did receive it [19]. Anecdotal evidences of steroid use in collapsing GN is there without any definitive guidelines [20].

## 4. Conclusions

Kidney biopsy needs to be strongly considered, when there is clinical evidence of glomerulus involvement. Gene testing for APOL-1 should be performed if the patients are of African descent and are having nephrotic-range proteinuria with rapidly progressive renal failure. More evidence is needed through larger studies in terms of establishing prognostic markers, treatments, long-term effects of COVID-19 on the kidneys, and early interventions to improve outcomes.

## Figures and Tables

**Table 1:** Admission labs with normal reference value.

	Value	Reference range
White blood cell count (10^9^/L)	12.6	4.5 to 11.0
Hemoglobin (g/dL)	9.2	13.5–14.5
Platelets (10^9^/L)	356	150–400
Sodium (mmol/L)	135	135–145
Potassium (mmol/L)	4.0	3.5–5.1
Carbon dioxide (mmol/L)	20	24
BUN (mg/dL)	52	7–20
Creatinine (mg/dL)	3.37	0.9–1.3
eGFR (mL/min)	23	>60
Albumin (g/dL)	3.5	4
Total protein (g/dL)	8.1	6–8.3
Creatinine kinase (U/L)	996	39–308
C-reactive protein (mg/dl)	12.5	0.0–0.60
Ferritin (ng/ml)	3624	26–388
Interleukin-6 (pg/ml)	104.5	0.0–15.5
UPCR (g/g)	15.8	0.0–.02
RBC on microscopy	>100/hpf	<3/hpf

**Table 2:** Renal parameters from admission to 6-week follow-up.

Laboratory value	Admission	Peak	Discharge	6 weeks after discharge
Creatinine	3.37	6.16	6.46	2.4
BUN	52	72	60	34
Urine protein/creatinine ratio	15.8	—	—	—
Hematuria	>100/hpf	—	—	—

## Data Availability

Data were obtained from the PubMed and Google Scholar database. The authors declare the data supporting the fundings of the study are available within the article.
